# EPSVR and EPMeta: prediction of antigenic epitopes using support vector regression and multiple server results

**DOI:** 10.1186/1471-2105-11-381

**Published:** 2010-07-16

**Authors:** Shide Liang, Dandan Zheng, Daron M Standley, Bo Yao, Martin Zacharias, Chi Zhang

**Affiliations:** 1 School of Engineering and Science, Jacobs University Bremen, Campus Ring 1, D-28759 Bremen, Germany; 2 Department of Radiation Oncology, Massey Cancer Center, Virginia Commonwealth University, Richmond, VA, 23298, USA; 3 Systems Immunology Lab, Immunology Frontier Research Center, Osaka University, Suita, Osaka, 565-0871, Japan; 4 Physics Department, Technical University Munich, James Franck Str., D-85747 Garching, Germany; 5 School of Biological Sciences, University of Nebraska, Lincoln, NE, 68588, USA

## Abstract

**Background:**

Accurate prediction of antigenic epitopes is important for immunologic research and medical applications, but it is still an open problem in bioinformatics. The case for discontinuous epitopes is even worse - currently there are only a few discontinuous epitope prediction servers available, though discontinuous peptides constitute the majority of all B-cell antigenic epitopes. The small number of structures for antigen-antibody complexes limits the development of reliable discontinuous epitope prediction methods and an unbiased benchmark to evaluate developed methods.

**Results:**

In this work, we present two novel server applications for discontinuous epitope prediction: EPSVR and EPMeta, where EPMeta is a meta server. EPSVR, EPMeta, and datasets are available at http://sysbio.unl.edu/services.

**Conclusion:**

The server application for discontinuous epitope prediction, EPSVR, uses a Support Vector Regression (SVR) method to integrate six scoring terms. Furthermore, we combined EPSVR with five existing epitope prediction servers to construct EPMeta. All methods were benchmarked by our curated independent test set, in which all antigens had no complex structures with the antibody, and their epitopes were identified by various biochemical experiments. The area under the receiver operating characteristic curve (AUC) of EPSVR was 0.597, higher than that of any other existing single server, and EPMeta had a better performance than any single server - with an AUC of 0.638, significantly higher than PEPITO and Disctope (*p-value *< 0.05).

## Background

Antigenic epitopes are regions of protein surface that are preferentially recognized by antibodies. Prediction of antigenic epitopes can help during the design of vaccine components and immuno-diagnostic reagents, but predicting effective epitopes is still an open problem in bioinformatics. Usually, B-cell antigenic epitopes are classified as either continuous or discontinuous. The majority of available epitope prediction methods focus on continuous epitopes [1-12].

Although discontinuous epitopes dominate most antigenic epitope families [13], due to their computational complexity, only a very limited number of prediction methods exist for discontinuous epitope prediction: CEP [14], DiscoTope [15], PEPITO [16], ElliPro [17], SEPPA [18], EPITOPIA[19,20] and our previous work, EPCES [21]. All discontinuous epitope prediction methods require the three-dimensional structure of the antigenic protein. The small number of available antigen-antibody complex structures limits the development of reliable discontinuous epitope prediction methods and an unbiased benchmark set is very much in demand [21,22].

In this work, we developed an antigenic Epitope Prediction method by using Support Vector Regression (EPSVR) with six attributes: residue epitope propensity, conservation score, side chain energy score, contact number, surface planarity score, and secondary structure composition. Further improvement was achieved by incorporating consensus results from a meta server, EPMeta, that we constructed using multiple discontinuous epitope prediction servers. The prediction accuracy was validated by an independent test set, in which antigens did not have available antibody-complex structures and epitopes were derived from various biochemical experiments.

## Results

### Prediction for the training set

Using the training procedure (see Methods), we obtained the optimized SVR parameters (i.e., *c*, *g*, and *p*). When *c *= 2^-6^, *g *= 2^-5^, *p *= 2^-3^, the mean value of the AUC for the 48 targets in the training set reached a maximum of 0.670 in the leave-one-out test. As a comparison, the mean AUC value was 0.644 when using EPCES, whose residue interface propensity was derived from the other 47 targets using the same leave-one-out procedure. The improvement of EPSVR could be attributed to the machine learning method because EPSVR and EPCES use the same six scoring terms. In another study, Rubinstein *et al*. applied support vector classifier (EPITOPIA) to predict B-cell epitopes and obtained a mean AUC value of 0.65 for a similar non-redundant set of 47 antigen-antibody complex structures in cross validation [19]. Our algorithm showed slightly better performance for a somewhat different training set.

### Prediction for the test set

We applied our algorithm, with optimally trained parameters, to the independent test set, and achieved a mean AUC value of 0.597, which was lower than that of the training set. Nevertheless, 6 out of 19 targets were predicted with an AUC value greater than 0.7. Here, we note that the epitopic residues of antigens in the test set were identified by point mutations, overlapping peptides, and ELISA, which are not as accurate as that based on crystal structures.

Six antigens in test proteins (PDB IDs: 1eku, 1av1, 1al2, 1jeq, 2gib, and 1qgt) contained multiple chains, but we only used a single chain, where the experimental antigenic epitope was located, for prediction. If the whole protein was used for prediction, the mean AUC value of the 6 proteins decreased from 0.672 to 0.623. When using the single chain in a multimer, we excluded the other chains from the prediction model. When using multiple chains, we considered all chains, and the total number of surface residues was counted for the intact complex structure. Unlike antigenic epitopes, the interfaces of protein-protein complexes, especially non-transient complexes, are usually more hydrophobic and conserved than protein surfaces; this makes the exposed protein-protein interfaces relatively easily to distinguish from both the antigenic epitopes and other protein surfaces. In other words, the prediction task for a single chain protein that has both protein-protein binding interfaces and an epitope is easier than that of a complete protein complex.

### Comparison with other algorithms

**Figure 1:**
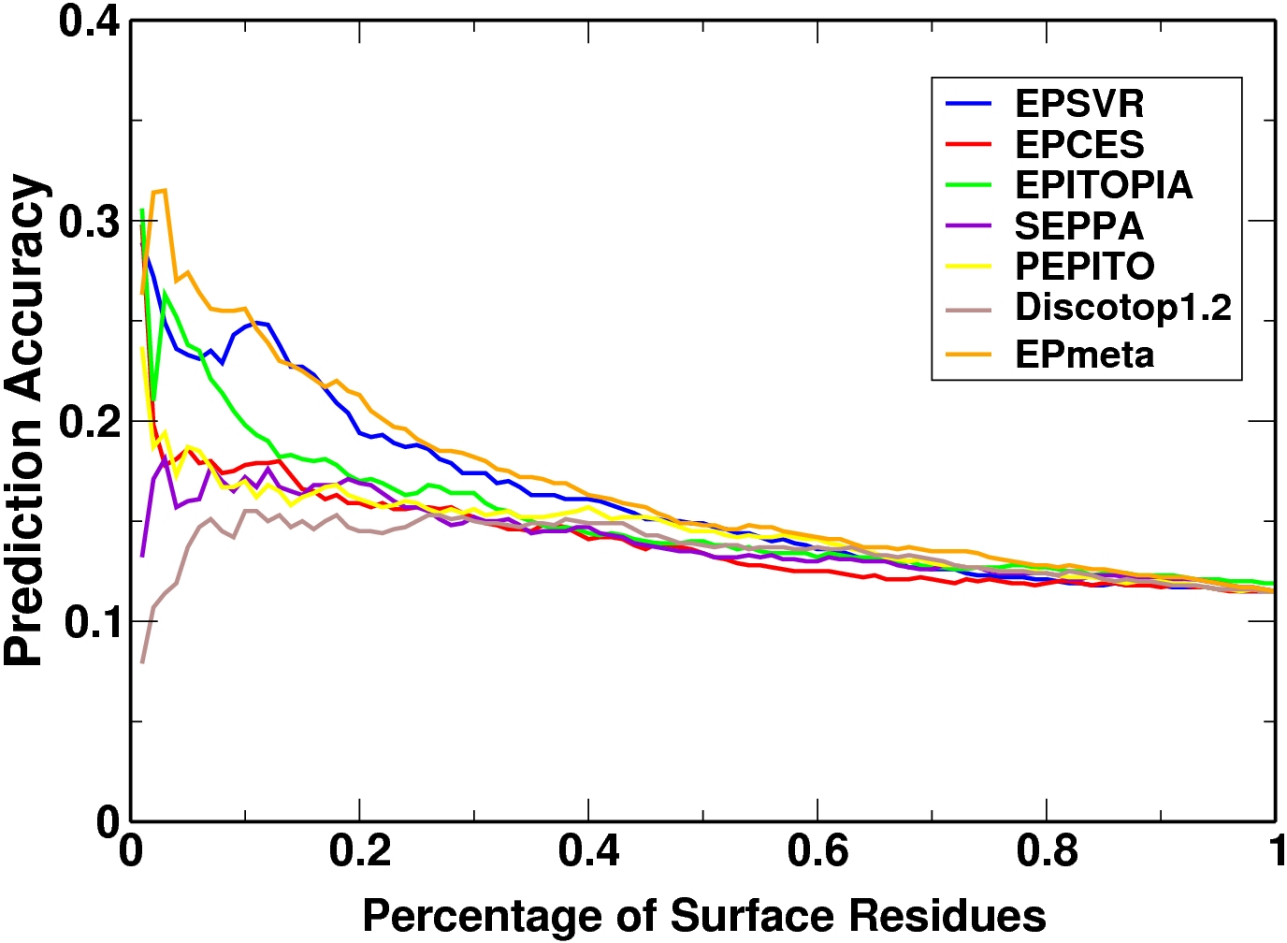
**Prediction accuracy of six antigenic epitope prediction servers and meta server on 19 independent testing proteins**. (EPITOPIA failed to assign scores to the antigenic residues of protein 1jeq and the prediction accuracy was averaged by the other 18 proteins.)

The limited number of available antigen-antibody complex structures is one of the main obstacles in developing and testing of antigenic epitope prediction algorithms. Current prediction algorithms use most or all of the available antigen-antibody complex structures as training data. An independent test set would be valuable to evaluate new prediction algorithms. Here, we compiled 19 proteins with epitope information derived from experimental methods other than crystal structures. With this independent test set, we compared our method with five recently developed algorithms: DiscoTope1.2 [15], PEPITO [16], SEPPA [18], EPITOPIA [19], and EPCES [21]. The mean AUC value of EPSVR was 0.597, and that of the others was 0.567, 0.570, 0.576, 0.579, and 0.586, respectively. Although EPSVR had the best performance, according to the pairwise t-student test, the differences between EPSVR and other servers were not statistically significant (*p-value *> 0.05), partly due to the small number of testing proteins. When 10% of surface residues were returned as predicted epitopic residues by each server, the accuracy was 24.7%, 17.8%, 15.5%, 17.0%, 17.2%, 18.8% for EPSVR, EPCES, DiscoTope1.2, PEPITO, SEPPA, and EPITOPIA, respectively (Fig. 1). In a random prediction, the accuracy was 11.5% (the mean value of number of epitopic residues/the number of surface residues for the 19 proteins). As a specific example, the antigenic epitope of 1jeq was located on a small C-terminal domain and the connection loop was invisible in the crystal structure. The whole C-terminal domain was ignored by EPITOPIA and its AUC value was set to 0 because it had no prediction for that section at all. Excluding 1jeq, EPITOPIA achieved the best average AUC value of 0.611 for the remaining 18 targets, while EPSVR achieved an accuracy of 0.591 for the same 18 targets. Nevertheless, EPSVR had better prediction results for targets with a relatively high percentage of mapped antigenic residues, which made a significant contribution to the average prediction accuracy. As a result, EPSVR showed overall higher prediction accuracy than EPITOPIA, as shown in Fig. 1.

### Meta method

As shown above, the prediction accuracy of the different methods decreased in the order of EPSVR, EPCES, EPITOPIA, SEPPA, PEPITO, and Discotope1.2. For the meta server, the basic idea was that a surface residue is predicted as an epitopic residue if two or more single servers voted for it. In this naive sense, the mean AUC values of the 19 testing proteins was calculated to be 0.562, 0.618, 0.627, 0.621, and 0.612 predicted by the top 2, 3, 4, 5, and 6 servers, respectively. To adopt a more sophisticated strategy, the top 25% of surface residues were returned as predicted epitopic residues by EPSVR, EPCES, and EPITOPIA. When the number of the predicted residues was increased from 25% to 50%, from 50% to 75%, and from 75% to 100%, SEPPA, PEPITO, and Discotope1.2 were, respectively, included in the voting. For example, the new antigenic residues predicted by EPSVR, EPCES, EPITOPIA, and SEPPA were added to the top 25% residues predicted by EPSVR, EPCES, and EPITOPIA. The prediction started with 1% of the surface residues for each of the four servers and increased in steps of 1% until 50% of surface residues were predicted as antigenic residues. Then we added PEPITO and used five servers to predict the top 50%~75% surface residues and so on. With this method, we achieved a mean AUC value of 0.638, which is higher than all single servers, especially, Discotope1.2 and PEPITO (*p*-value < 0.05). The reason that we used this strategy to integrate the various predictions results from our finding that a single server had better prediction accuracy when only a small fraction of the surface residues were predicted as epitopic residues (Fig. 1). If 50% of surface residues, for example, were predicted as epitopic residues by the meta server, the prediction accuracy was 14.4% for the meta server with a voting set including EPSVR, EPCES, and EPITOPIA, where each server output the top 51% surface residues as candidates of antigenic residues. As a comparison, the prediction accuracy was slightly higher (15.3%), if the meta server also returned 50% of the surface residues as eptitopic residues, but got votes for those returned residues from all of the six servers, where each server output their own top 32% surface residues as candidates of epitopic residues. This approach can be summarized in the following pseudo-code:

N = the total number of surface residues;

E = the number of predicted epitopic residues;

if E ≤25% * N then,

return Predictor (0, E, EPSVR, EPCES, EPITOPIA);

else if E > 25%*N AND E ≤50%*N then,

return Predictor (R25, E, EPSVR, EPCES, EPITOPIA, SEPPA);

else if E > 50% AND E ≤75%*N then,

return Predictor (R50, E, EPSVR, EPCES, EPITOPIA, SEPPA, PEPITO);

else if E > 75% AND E ≤100%*N then,

return Predictor(R75, E, EPSVR, EPCES, EPITOPIA, SEPPA, PEPITO, Discotope1.2)

endif.

R*p *= *p*% of surface residues already predicted as epitopic residues;

Function Predictor(R*p*,E,SERVER1,SERVER2,SERVER3,...)

Begin

set the prediction of each single server to 0;

do {

Increase the prediction of each single server at the step of 1%;

Collect residues predicted by at least two of the servers;

} While(R*p *+ collected epitopic residues other than R*p *< E);

Return total epitopic residues;

END.

Although EPSVR and EPCES used the same six scoring terms, we found that it was necessary to include both of them in the meta server. When we used a voting server set including only EPCES, EPITOPIA, and SEPPA (i.e. excluding EPSVR) the average AUC value decreased to 0.587 for the test set. The average AUC value predicted by EPSVR, EPITOPIA, and SEPPA (0.611) was also lower than that predicted by EPSVR, EPCES, and EPITOPIA in the standard procedure (0.618). We also tried to increase the threshold of votes from two to three for a voting server set, but the results did not improve.

## Conclusions

We introduced a SVR method to integrate six attributes for discontinuous epitope prediction and a server, EPSVR, which can be accessed online. The AUC of EPSVR is 0.597, which is higher than that of any other existing single server. Although they used the same scoring functions, EPSVR exhibited improved performance over EPCES. This was attributed to the fact that EPSVR searched the six-dimensional parameter space of all scores more broadly than the voting method we previously used. Furthermore, a meta server, EPMeta, combining EPSVR and the other existing single servers together, had an AUC value of 0.638, which is higher than any single server, especially, Discotope and PEPITO. We also found that the use of both EPSVR and EPCES, which use the same 6 scoring terms, resulted in a higher performance for EPMeta than if only one was used.

## Methods

### Datasets

#### Training set

The training set was gathered and screened from three protein data sets: 1) 22 antigen-antibody complexes and their unbound structures from protein docking Benchmark 2.0 [23]; 2) 59 representative antigen-antibody complexes compiled by Ponomarenko and Bourne [22]; 3) 17 antigen-antibody complex structures released between February 2006 and October 2008 with available unbound antigen structures, which was the test set in our previous work [21]. Any antigen-antibody complex was not used as a training structure if its antigen had no available unbound structure because unbound structures were required for prediction. A complex structure was also not used if its antigenic epitope consisted of amino acid residues located on multiple chains. A complex was included if the sequence identity between its antigen and all antigens from the other complex structures was less than 35% following local sequence alignment. For an antigen with a sequence identity in the range of 35~50%, we accepted the antigen-antibody complex if the binding topology was not the same as its homologous complex. For an antigen with more than one antigenic epitope, only one was used in order to avoid confusion in subsequent application of support vector regression methods. As a result, a total of 48 complexes and their unbound structures meeting the above criteria were used as a training set, available for download at http://sysbio.unl.edu/services/.

#### Test set

The test set was curated from 293 entries of the Conformational Epitope Database [24] (CED, Release 0.03) with the following criteria. We only considered entries that had unbound antigen structures, but no complex structures. Multiple entries with the same antigen structure were combined and considered as one target, and antigenic residues from multiple entries were mapped onto one protein structure. The sequence identity between any two selected proteins was also required to be less than 35%. All selected antigens were also screened against the rest of CED database and our training set; the sequence identity between a selected antigen and other antigens with complex structures in the CED or in the training set was less than 35%. A total of 22 antigenic proteins in the CED met all the above criteria; these were: 1www, 1hgu, 1eku, 1mbn, 1av1, 1pv6, 1al2, 2gmf, 1a7c, 1y8o, 1og5, 1jeq, 1dab, 1w7b, 1ly2, 1rec, 1nu6, 2b5i, 2gib, 1p4t, 1xwv, and 1qgt. Three antigenic proteins, 1www, 1hgu, and 1xwv, were excluded since they had multiple antibody-binding sites and the mapped antigenic residues were evenly distributed on the protein surfaces. Therefore, the final test set contained 19 antigen structures, available at http://sysbio.unl.edu/services/.

### Support Vector Regression

#### Training procedure

For each surface patch, the number of epitopic residues could be any integer value between 0 and the patch size (20 for this work), and each surface patch had six Support Vector Regression (SVR) attributes, which were calculated with the six scoring terms: residue epitope propensity, conservation score, side chain energy score, contact number of the central residue, surface planarity score, and secondary structure composition. The residue epitope propensity, conservation score, and side chain energy score were calculated at the residue level and averaged over all residues in the patch. The six scores and the number of observed epitopic residues in the patch were scaled to 0~1. The six scoring terms were the same as used in our previous work [21], where details can be found, and hence, we describe them here briefly. The residue epitope propensity was computed as the product of the normalized solvent accessible surface of the residue and the logarithm ratio of the epitopic area to the rest area for a given residue. The conservation score was measured by the difference between the self-substitution score in the position-specific substitution matrix generated from PSIBLAST and the diagonal element of BLOSUM62 for the residue type. Epitopic residues are not as conserved as other surface residues. The energy function to calculate side chain energy score was optimized so that the native structure of a residue was predicted energetically favorable compared with other residue types at each position of the training proteins. Those residues of high energy could be responsible for protein binding; we previously used the similar terms for protein-protein interface prediction. The rest three terms: contact number of the central residue, surface planarity score, and secondary structure composition, have already been used for antibody binding site prediction by others.

All SVR parameters were optimized by a grid search (*c *= 2^-10~-1^, *g *= 2^-12~-3^, and *p *= 2^-5~-2^); and for each grid point of triplets, a leave-one-out procedure was applied to evaluate the trained SVR model. Specifically, the patch score of each surface patch for a target in the training set was predicted by the SVR model trained with the other 47 antigen-antibody complexes, from which the residue epitope propensity score was also derived. After this procedure was repeated 48 times, the mean AUC value of 48 predictions represents the performance of the current grid point for SVR parameters. The triplet of parameters that reached the highest value of mean AUC was chosen and used for the test set, and the final support vector machine model was trained with all 48 targets.

#### Prediction procedure

A surface patch is defined as a central surface residue and its 19 nearest surface neighbors in space, where a surface residue is defined if the relative accessibility of its side chain is greater than 6% with probe radius = 1.2Å. First, we searched for all surface residues and enumerated all surface patches of a given antigen structure, and calculated their six SVR attributes. For each surface patch, we predicted the number of putative epitopic residues by the trained SVR model. Here, a patch score was defined as the fraction of the number of putative epitopic residues to the total number of amino acid residues in the patch, i.e. 20. One surface residue was assigned a residue score by averaging patch scores of all patches in which this amino acid residue is included. Finally, we sorted surface residues according to their residue scores and the top-ranked ones were considered as epitopic residues. The assumption here is that a residue frequently appearing in top-scoring patches is likely an epitopic residue.

Patch analysis was used in all existing B-cell discontinuous epitope studies. In the examples of EPCES and EPITOPIA, a patch score was derived by averaging the scores of all residues in the patch, and the central residues of top scored patches were predicted as epitopic residues. However, the value of the patch score was actually correlated with the number of epitopic residues in the patch rather than the central residue. Here, we used SVR to predict the number of epitopic residues in a surface patch and residues frequently located in the top scored patches were predicted as epitopic residues. For this case, the SVR model is more suitable than a support vector classifier. In this study, we used an SVR package, called LIBSVM, obtained from http://www.csie.ntu.edu.tw/~cjlin/libsvm.

### Evaluation methods

Prediction accuracy was defined as the ratio of the number of correctly predicted epitopic residues to the number of all predicted epitopic residues. The area under the receiver operating characteristic curve (AUC) was used as the primary evaluation metric. To obtain the ROC curve, we increased the number of predicted residues in steps of 1% of total surface residues. A java program available at http://pages.cs.wisc.edu/~richm/programs/AUC/ was used to calculate the AUC.

## Availability and requirements

**Project name**: Prediction of Antigenic Epitopes.

**Project home page**: Both servers are available at http://sysbio.unl.edu/services.

**Operating system**: Platform independent.

**Programming language**: c++ and perl.

**Other requirements**: no.

**License**: free for academics.

**Any restrictions to use by non-academics**: license needed.

## Authors' contributions

SL designed the study, implemented the algorithm and drafted the manuscript. DZ and DMS helped prepare the data and draft the manuscript. BY and CZ built the web servers. MZ supervised the study. All authors read and approved the final manuscript.
